# Benchmarking drug–drug interaction prediction methods: a perspective of distribution changes

**DOI:** 10.1093/bioinformatics/btaf569

**Published:** 2025-10-14

**Authors:** Zhenqian Shen, Mingyang Zhou, Yongqi Zhang, Quanming Yao

**Affiliations:** Department of Electrical Engineering, Tsinghua University, Beijing, 100084, China; Department of Electrical Engineering, Tsinghua University, Beijing, 100084, China; Thrust of Data Science and Analytics, The Hong Kong University of Science and Technology (Guangzhou), Guangdong, 511453, China; Department of Electrical Engineering, Tsinghua University, Beijing, 100084, China; State Key Laboratory of Space Network and Communications, Tsinghua University, Beijing, 100084, China

## Abstract

**Motivation:**

Emerging drug–drug interaction (DDI) prediction is crucial for new drugs but is hindered by distribution changes between known and new drugs in real-world scenarios. Current evaluation often neglects these changes, relying on unrealistic i.i.d. split due to the absence of drug approval data.

**Results:**

We propose DDI-Ben, a benchmarking framework for emerging DDI prediction under distribution changes. DDI-Ben introduces a distribution change simulation framework that leverages distribution changes between drug sets as a surrogate for real-world distribution changes of DDIs, and is compatible with various drug split strategies. Through extensive benchmarking on 10 representative methods, we show that most existing approaches suffer substantial performance degradation under distribution changes. Our analysis further indicates that large language model (LLM)-based methods and the integration of drug-related textual information offer promising robustness against such degradation. To support future research, we release the benchmark datasets with simulated distribution changes. Overall, DDI-Ben highlights the importance of explicitly addressing distribution changes and provides a foundation for developing more resilient methods for emerging DDI prediction.

**Availability and implementation:**

Our code and data are available at https://github.com/LARS-research/DDI-Bench.

## 1 Introduction

With the rapid development of drug discovery, numerous emerging drugs are being developed to treat various diseases. As these drugs contain novel chemical substances with unknown pharmacological risks, it is crucial to conduct emerging drug–drug interaction (DDI) prediction to identify not only potential adverse interactions but also opportunities for effective combination therapies. In clinical experiments, measuring and verifying DDIs are extremely time-consuming and expensive, which motivates recent development of computational methods for the problem of DDI prediction. Currently, the research on computational methods for DDI prediction problem has been more and more prevalent, and different types of machine learning techniques have been used for that problem, including feature-based methods (Rogers and Hahn 2010, Ryu *et al.* 2018, Liu *et al.* 2022), embedding-based methods (Karim *et al.* 2019, Yao *et al.* 2022), graph neural network (GNN)-based methods (Zitnik *et al.* 2018, Lin *et al.* 2021, Yu *et al.* 2021, Zhang *et al.* 2023), graph transformer-based methods (Su *et al.* 2024, Chen *et al.* 2024), and large language model (LLM)-based methods (Zhu *et al.* 2023, Xu *et al.* 2024, Abdullahi *et al.* 2025).

**Figure 1.**
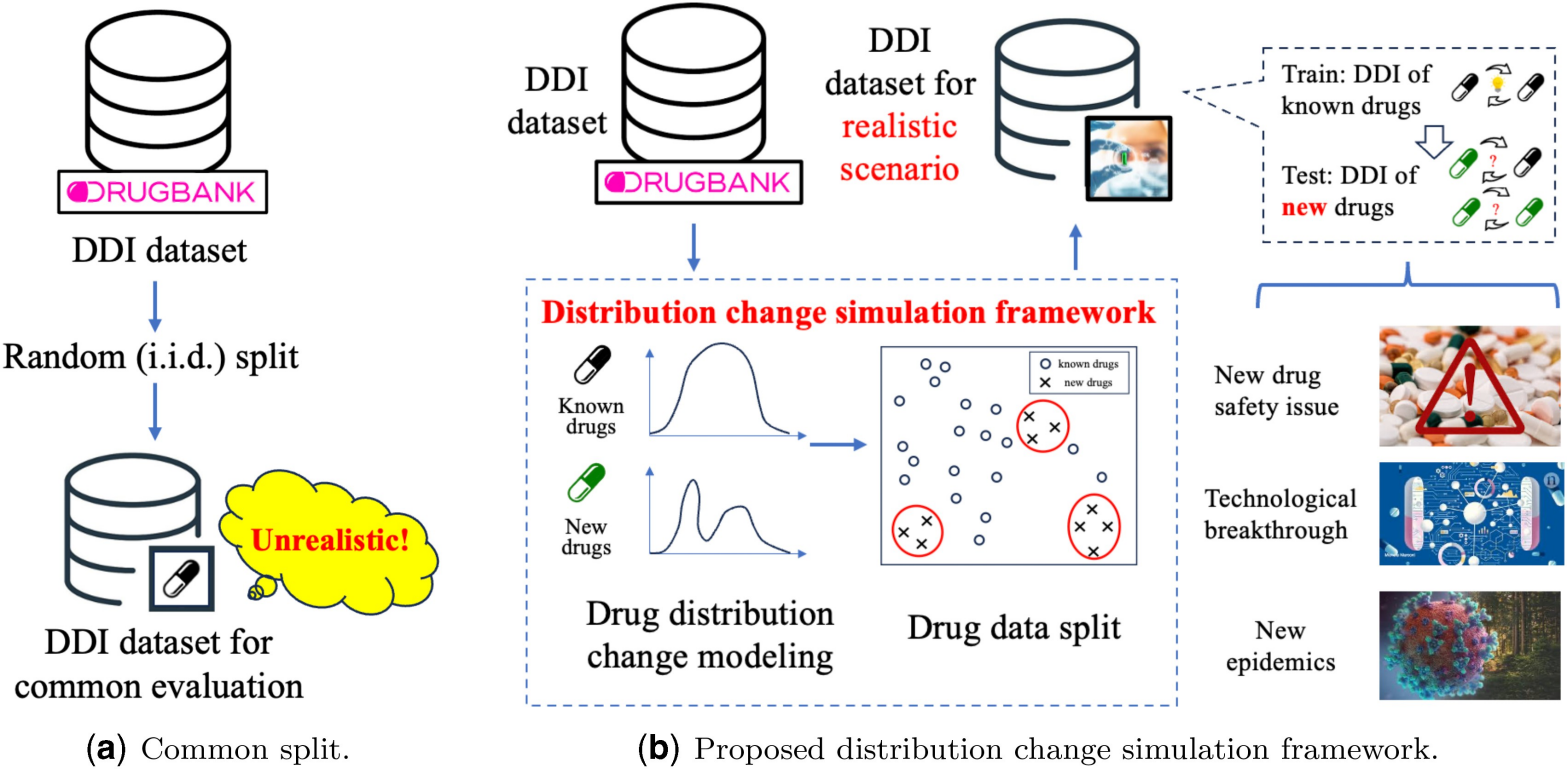
Comparison between common DDI data split and proposed distribution change simulation framework for emerging DDI prediction evaluation.

In this work, we focus on benchmarking for emerging DDI prediction in a perspective from distribution changes, attempting to reduce the mismatching of existing DDI prediction evaluation and realistic drug development scenarios. Current evaluation frameworks for emerging DDI prediction methods inadequately address the phenomenon of distribution changes inherent in real-world data, primarily due to following reasons: firstly, current widely-used DDI datasets [e.g. Drugbank (Tatonetti *et al.* 2012), TWOSIDES (Wishart *et al.* 2018)] lack information on approval timelines of drugs, making it hard to directly incorporate drug distribution changes into the evaluation framework. Secondly, owing to the lack of time information for drugs, most of the existing emerging DDI prediction methods implicitly assume that known drugs and new drugs follow the same distribution (Liu *et al.* 2022, Zhang *et al.* 2023), dividing drugs into known and new drug sets in an i.i.d. manner (Fig. 1a). They neglect the phenomenon of distribution changes, i.e. inherent in realistic drug development process, which makes their evaluation results questionable. In order to evaluate existing DDI prediction methods in a scene closer to real-world scenarios, it is necessary to simulate the distribution changes between known and new drugs.

To fulfill the gap, we propose DDI-Ben, a benchmark for emerging DDI prediction that investigates existing computational methods in a perspective from drug distribution changes. We first introduce a distribution change simulation framework (Fig. 1b), i.e. compatible with different drug split schemes to replicate distribution changes in real-world DDI data. We conduct extensive experiments on representative emerging DDI prediction methods, ranging from simple MLP to recent ones based on LLMs. We observe a significant drop in the performance of existing methods on emerging DDI prediction tasks with distribution change introduced. It is also found that LLM, drug-related textual information can be key factors to alleviate the negative impact of that performance drop. In summary, our main contributions are as follows:

- DDI-Ben proposes a distribution change simulation framework that uses distribution changes between drug sets as a surrogate to simulate distribution changes in emerging DDI prediction problem. The framework is compatible with various drug split strategies, which can reflect the distribution changes in real-world scenarios.
- Through benchmarking evaluations on emerging DDI prediction, DDI-Ben demonstrates that most existing methods lack robustness against distribution changes. Our analysis suggests that developing LLM-based approaches and incorporating drug-related textual information can help mitigate performance degradation under such conditions.
- We also release emerging DDI prediction dataset with simulated distribution changes used in our benchmark.

## 2 Emerging DDI prediction task description

**Figure 2.**
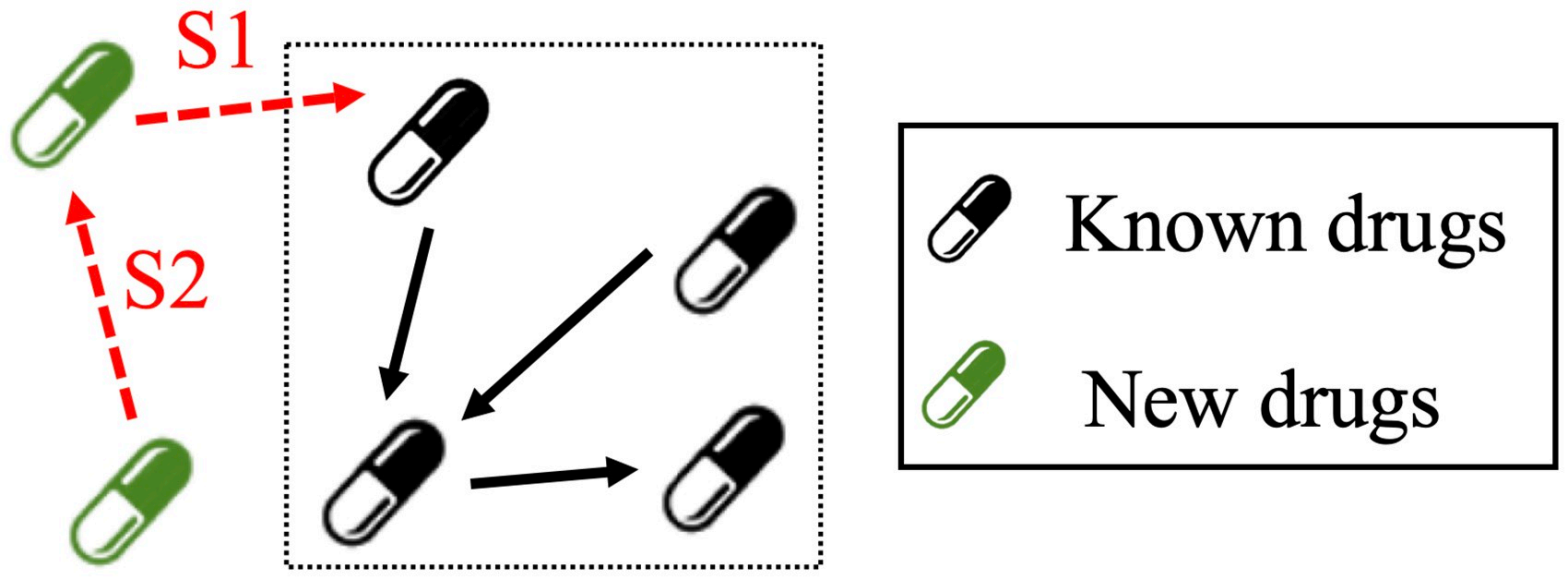
Emerging DDI prediction task description.

Assume the drug set is D and the possible interaction set between drugs is R. The problem of DDI prediction is to learn a predictor p:D×D→R that can accurately predict the interaction type r∈R between drugs (u,v)∈D×D. To conduct emerging DDI prediction evaluation, drug set D is usually divided into two sets: known drug set $D_{k}$ and new drug set $D_{n}$. We mainly focus on the DDI prediction relevant to emerging (new) drugs, including two types of tasks (see Fig. 2). The S1 task is to determine the DDI type between a known drug and a new drug. The S2 task is to determine the DDI type between two new drugs. As mentioned above, the distribution changes between training and test DDIs are neglected in existing DDI prediction evaluation settings, which makes their evaluation results unreliable in realistic drug development scenarios.

## 3 Distribution change simulation framework

In emerging DDI prediction, distributional changes between training and test data are critical issues, which primarily stems from distribution changes between known and new drugs (Wishart *et al.* 2018, Celebi *et al.* 2019). However, existing DDI datasets do not provide approval time information for drugs, making it hard to directly distinguish known and new drugs. To address this problem, we model distribution changes between known drug set and new drug set as a surrogate to simulate distribution changes.

In this section, we first introduce a distribution change simulation framework that can reflect realistic scenarios in emerging DDI prediction, with an analysis for understanding of that framework. Based on observation of real-world data, we propose cluster-based distribution change modeling for DDI benchmarking evaluation. Finally, we provide a comparison among different drug split strategies and evaluate their consistency with realistic drug split scheme.

### 3.1 Drug distribution change as surrogate

In order to better capture distribution changes in realistic drug development process and conduct emerging DDI prediction evaluation, we propose a distribution change simulation framework (Fig. 1b), where we use drug distribution changes as a surrogate to simulate the distribution changes in emerging DDI prediction problem. It mainly contains steps as follows:

- Drug distribution change modeling: Introduce a measurement to model the distribution change between known drug set $D_{k}$ and new drug set $D_{n}$. Use that distribution change as a surrogate of distribution change between training and test DDIs in emerging DDI prediction problem.
- Drug data split: Conduct a split of drugs into known and new drug sets, then obtain DDI dataset for emerging DDI prediction evaluation.

For common DDI data split (as shown in Fig. 1a) in most existing works (e.g. Liu *et al.* 2022, Yao *et al.* 2022, Zitnik *et al.* 2018, Zhang *et al.* 2023), the phenomenon of distribution change is not considered, leading to unrealistic DDI evaluation results. Through the distribution change simulation framework, we can better simulate the distribution changes and conduct emerging DDI prediction evaluation in a scene closer to real-world scenarios.

### 3.2 Understanding

**Figure 3.**
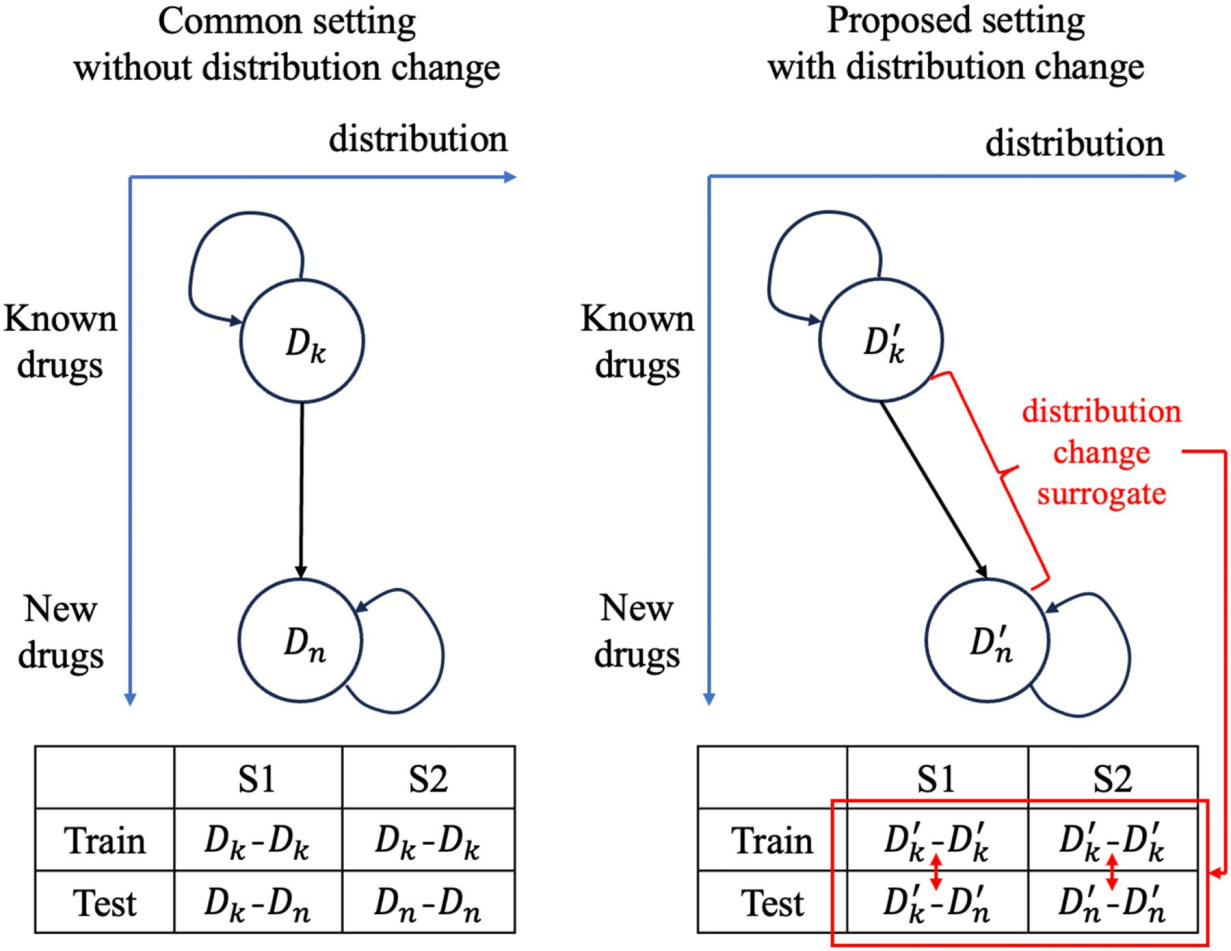
Illustration of DDI data split.

The split of DDI data in different settings in S1 and S2 tasks is illustrated in Fig. 3. In common setting, there is no distribution change between known drug set $D_{k}$ and new drug set $D_{n}$. The training and test DDIs for S1 and S2 tasks follow the same distribution, which does not conform with real-world drug development process. Compared with common setting, the proposed setting introduces the distribution change between known drug set $D_{k}^{\prime }$ and new drug set $D_{n}^{\prime }$ as a surrogate of the distribution changes between training and test DDIs for emerging DDI prediction evaluation. By incorporating these two different evaluation settings, we can more effectively benchmark the impact of distributional changes on DDI prediction methods under realistic scenarios.

### 3.3 Customized distribution change surrogate

**Figure 4.**
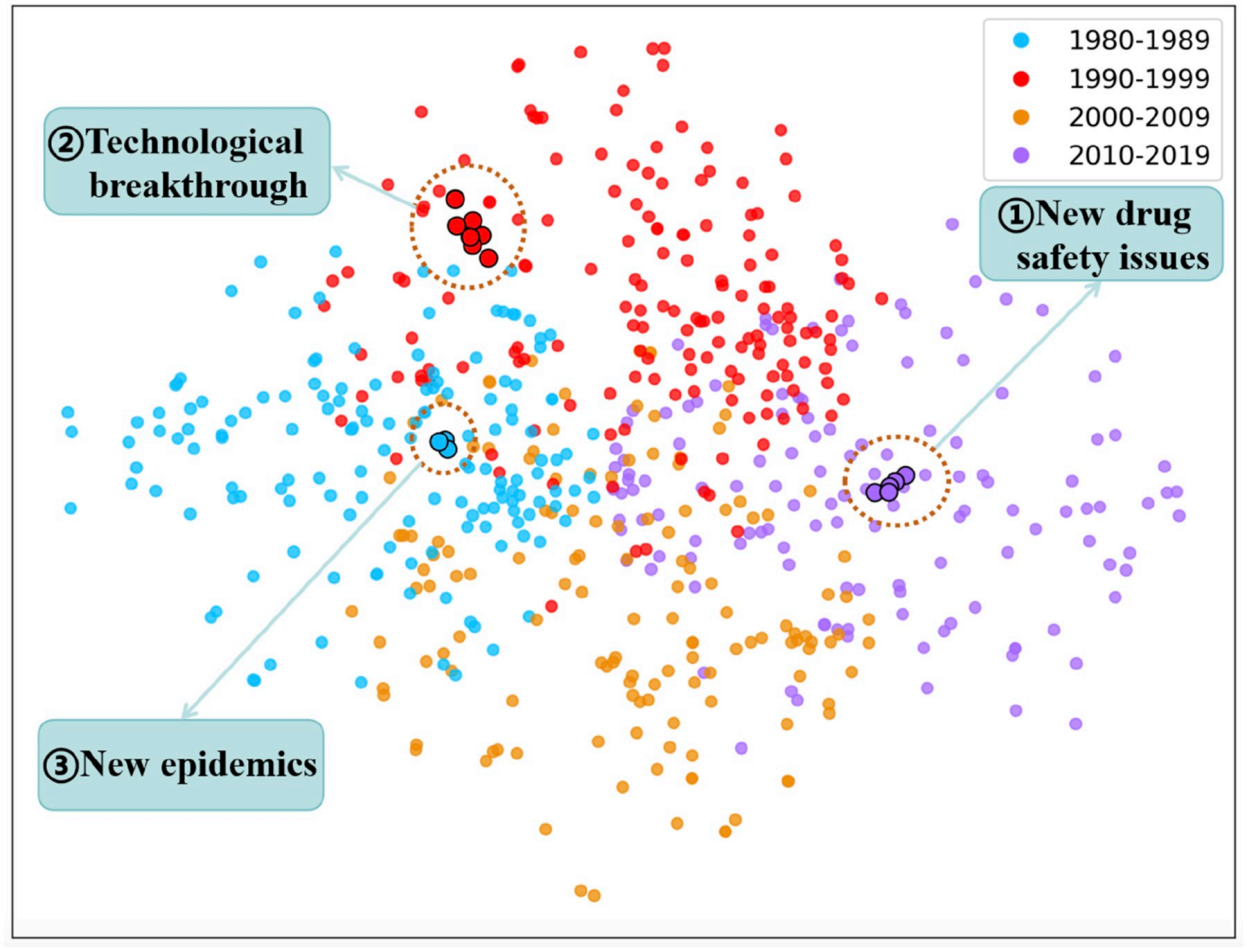
T-SNE illustration of drug distribution with their approval time in Drugbank dataset.

Following the above distribution change simulation framework, we first collect approximate approval times of a part of drugs in Drugbank dataset and visualize their distribution in the chemical space (as shown in Fig. 4). We can see that drugs developed in specific time periods demonstrate a clustering effect in the chemical space owing to various factors, such as (i) new drug safety issues (Rao and Knaus 2008), (ii) technological breakthrough for drug development (Cushman and Ondetti 1991), and (iii) new epidemics (Pawlotsky 2013).

Based on the above observation, we consider to design a customized cluster-based difference measurement to model the distribution changes between known and new drug set. Denote known and new drug set as $D_{k}$ and $D_{n}$. We define difference between two drug sets as $\gamma (D_{k},D_{n})=\max\{S(u,v),\forall u\in D_{k},v\in D_{n}\}$, where S(·,·) is similarity measurement of two drugs. Then we utilize parameter γ as a surrogate to control distribution changes between training and test DDIs in emerging DDI prediction evaluation. With the decrease of γ, the difference between known and new drug set will become more significant, leading to larger distribution changes between training and test DDIs.

Remark 1 .

The proposed cluster-based difference measurement is a reasonable surrogate under the proposed distribution change simulation framework. Additional factors that may lead to distributional changes between known and new drug sets could also be simulated and explored in future studies.

### 3.4 Comparison among different data split strategies

In this section, we provide comparison among different data split strategies as follows: (i) random split (Zitnik *et al.* 2018), (ii) drug frequency based split (Jamal *et al.* 2020), (iii) drug usage based split (Glorot *et al.* 2011), (iv) scaffold split (Yang *et al.* 2019) (v) LoHi (Steshin, 2023), (vi) SPECTRA (Ektefaie *et al.* 2024), (vii) DataSAIL (Joeres *et al.* 2025), and (viii) the proposed cluster-based split (Koh *et al.* 2021).

Table 1 shows the overall comparison among the eight data split strategies. LoHi and SPECTRA need to discard a part of data points in the split process, which is generally viewed as undesirable given the high cost of drug data collection. Only SPECTRA and the cluster-based split generate splits that involve controllable distributional changes, enabling the examination of how different DDI prediction methods perform under increasingly pronounced distribution changes.

**Table 1.** Comparison among different data split strategies.

Split strategy	Random	Drug frequence	Drug usage	Scaffold	LoHi	SPECTRA	DataSAIL	Cluster
Preserve all data	✓	✓	✓	✓	×	×	✓	✓
Controllable distribution change	×	×	×	×	×	✓	×	✓
Consistency with approval time	Low	Low	Low	Low	Medium	Low	Medium	High

**Figure 5.**
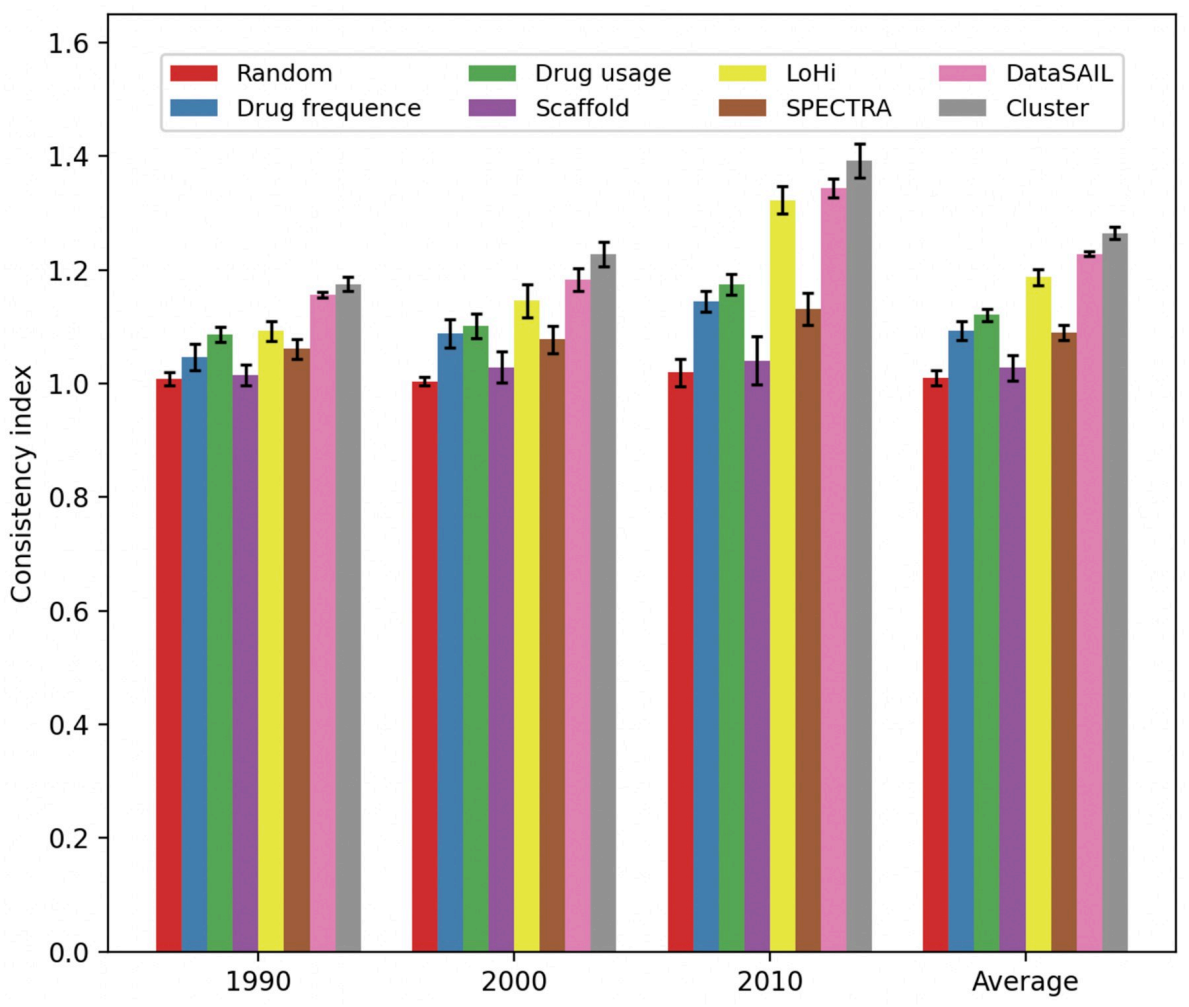
Figure for time threshold of the realistic drug split scheme w.r.t the consistency index of each drug split scheme.

To further assess the consistency between different split strategies and realistic data, we conducted verification experiments. Using the recorded approval times of a subset of drugs from the DrugBank dataset, we designated specific time points as thresholds to separate drugs into known and new categories, thereby constructing a realistic drug split scheme. For each threshold, we computed a consistency index between every drug split scheme and the realistic split, quantifying the degree of alignment for drug split strategies with real-world scenarios. The results, present in Fig. 5, demonstrate that the proposed cluster-based split consistently achieves the highest consistency index, indicating closer conformity to the realistic split. Compared with other split strategies, LoHi and DataSAIL are also relatively more consistent with realistic drug split scheme. Based on these results, we choose cluster-based split to simulate realistic distribution changes in benchmarking evaluation of emerging DDI prediction.

## 4 Instantiation of the proposed framework

In this section, we first instantiate the proposed distribution change simulation framework by drug split based on cluster-based difference measurement for emerging DDI prediction evaluation. The evaluated methods and evaluation metrics in benchmarking experiments are subsequently introduced.

### 4.1 Dataset and split

In DDI-Ben, we conduct experiments on two widely-used public DDI datasets: (i) Drugbank (Wishart *et al.* 2018), a multiclass DDI prediction dataset where each drug pair in this dataset is associated with one of 86 possible interaction types. (ii) TWOSIDES (Tatonetti *et al.* 2012), a multilabel DDI prediction dataset that records side effects between drugs. We keep 209 DDI types (occurrence frequency from 3000 to 6000) to ensure each DDI type corresponds to enough drug pairs for learning, and each drug pair may have multiple interactions among 209 DDI types.

The drug split based on cluster-based difference measurement is shown in Algorithm 1. Through the algorithm, the difference measurement between known and new drug set satisfies $\gamma (D_{k},D_{n})\leq \gamma_{0}$. Hence, we can control the distribution changes between known and new drug sets through adjustment of the parameter $\gamma_{0}$, where smaller $\gamma_{0}$ indicates more significant distribution changes. For common DDI evaluation setting, we randomly split drugs into known drug set $D_{k}$ and new drug set $D_{n}$. With known and new drug sets, we split DDI data for emerging DDI prediction evaluation. In both S1 and S2 tasks, training set includes all DDI triplets (u,r,v), where *u* and *v* are both in known drug set $D_{k}$. The test set in S1 task comprises DDI triplets (u,r,v), where one drug *u* is in known drug set $D_{k}$ and the other drug belongs to new drug set $D_{n}$. The test set in S2 task contains DDI triplets (u,r,v), where both drugs *u* and *v* are in new drug set $D_{n}$.

Algorithm 1. Drug split based on cluster-based difference measurement.

**Require:** The drug set *D*, the similarity measurement S(·,·).

1: Calculate the similarity between drug pairs in the dataset *D* based on measurement *S*.

2: Determine the distribution change parameter $\gamma_{0}$. Regard different drugs as nodes in a graph. For each drug pair in the dataset, if their similarity is larger than $\gamma_{0}$, we build connection between these two drugs.

3: Based on connections of drugs in the graph, we regard each connected component in the graph as a drug cluster. The drug graph is divided into *m* clusters $D_{1},D_{2},\ldots ,D_{m}$.

4: Randomly divide drug clusters into known drug set $D_{k}$ and new drug set $D_{n}$. **return** Known drug set $D_{k}$ and new drug set $D_{n}$.

### 4.2 Methods to be compared

We choose 10 representative methods to be compared in DDI-Ben. Their category and used side information are summarized in Table 2. MLP (Rogers and Hahn 2010) is the most classical feature-based method that designs a multi-layer perceptron to predict the DDI based on input drug fingerprints. MSTE (Yao *et al.* 2022) specially designs a knowledge graph embedding scoring function that can perform well for DDI prediction problem. Decagon (Zitnik *et al.* 2018) is representative GNN-based methods that consider to incorporate biomedical network into DDI prediction. SSI-DDI (Nyamabo *et al.* 2021) designs a GNN to model drug molecular graphs and predict DDI based on interaction between substructure of query drug pairs. MRCGNN (Xiong *et al.* 2023) presents a multi-relation graph contrastive learning strategy to better characteristics of rare DDI types based on drug molecular structures. EmerGNN (Zhang *et al.* 2023) is a GNN-based method that specially designs a flow-based GNN to predict emerging DDIs. SAGAN (Zhang *et al.* 2025) utilizes a transfer learning strategy to enhance the cross-domain generalization ability of GNNs on DDI prediction tasks. TIGER (Su *et al.* 2024) is a graph-transformer-based method that designs a dual channel graph transformer to both capture the structural information of drug molecular graphs and the information from biomedical network. TextDDI (Zhu *et al.* 2023) is a LLM-based method that first try to use LLM to predict DDI based on textual information of drugs and drug interactions. DDI-GPT (Xu *et al.* 2024) captures relevant information of query drugs from biomedical networks and uses biomedical LLM to enhance DDI prediction performance.

**Table 2.** Main characteristics about DDI prediction methods we compared.

Method	Categorization	Side information used
MLP (Rogers and Hahn 2010)	Feature based	Drug fingerprints
MSTE (Yao *et al.* 2022)	Embedding based	–
Decagon (Zitnik *et al.* 2018)	GNN based	Drug fingerprints, biomedical networks
SSI-DDI (Nyamabo *et al.* 2021)	GNN based	Drug structural information
MRCGNN (Xiong *et al.* 2023)	GNN based	Drug structural information
EmerGNN (Zhang *et al.* 2023)	GNN based	Drug fingerprints, biomedical networks
SAGAN (Zhang *et al.* 2025)	GNN based	Drug structural information
TIGER (Su *et al.* 2024)	Graph-transformer based	Drug structural information, biomedical networks
TextDDI (Zhu *et al.* 2023)	LLM based	Textual data of drugs and drug interactions
DDI-GPT (Xu *et al.* 2024)	LLM based	Textual data of drugs and drug interactions, biomedical networks

### 4.3 Evaluation metric

Following the common practices (Yu *et al.* 2021, Zhang *et al.* 2023), we use F1 (primary), accuracy, Cohen’s Kappa (Cohen 1960) as evaluation metrics in multiclass DDI prediction for Drugbank dataset. Following the evaluation of Tatonetti *et al.* (2012) and Zitnik *et al.* (2018) in multilabel DDI prediction for TWOSIDES dataset, we report the average of ROC-AUC (primary), PR-AUC, and accuracy in each DDI type. The experimental results for evaluation metric accuracy, Cohen’s Kappa on Drugbank and PR-AUC, accuracy on TWOSIDES are shown in Supplementary Material, available as supplementary data at *Bioinformatics* online.

## 5 Results

We conduct extensive experiments to evaluate emerging DDI prediction in the proposed DDI-Ben benchmark in this section. Through experiments in settings with and without distribution changes, it is observed that most of the existing DDI prediction methods have significant performance drop with distribution changes introduced, and LLM-based methods are the most robust type of method against this negative impact. We further analyse performance on different DDI types and control distribution changes through parameter γ, providing in-depth insights into the influence of distribution changes.

### 5.1 Performance analysis among representative methods

**Figure 6.**
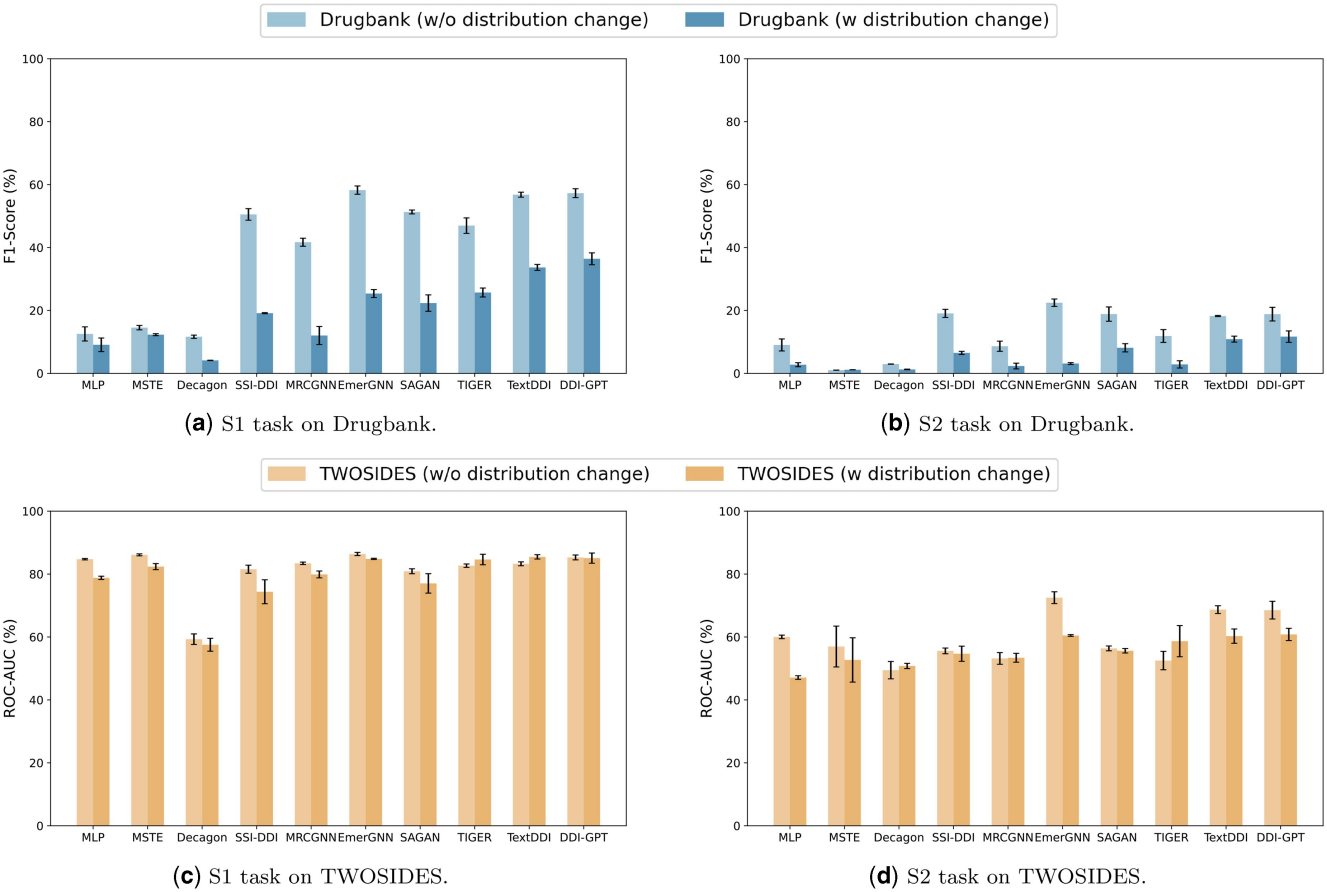
Performance comparison for different types of DDI methods in the settings with and without distribution change in S1–S2 tasks. Here, we utilize primary evaluation metric for each dataset (F1 for Drugbank and ROC-AUC for TWOSIDES).

We first conduct experiments on the two datasets with and without distribution changes introduced, and compare the performance of different DDI methods in two emerging DDI prediction tasks (S1–S2). The results are shown in Fig. 6.

For general comparison, we can see that injecting prior knowledge into method design may improve the performance. GNN-based and graph transformer-based methods outperform feature-based and embedding-based approaches, as they better leverage structural information from DDI graphs and side information from biomedical networks through neighborhood propagation. When comparing the two datasets, existing methods experience a more substantial performance decline on Drugbank when distribution changes are introduced, compared to their decline on TWOSIDES. This discrepancy can be attributed to the higher difficulty of multiclass prediction in the Drugbank dataset, where the larger number of classes increases the likelihood of misclassification under distribution changes.

### 5.2 Revealing factors that can alleviate the negative impact of distribution change

From the experimental results in Fig. 6, it can be seen that almost all of the evaluated methods exhibit performance degradation under distribution changes. However, the extent of performance degradation varies. Specifically, EmerGNN achieves the highest performance in the existing setting without distribution changes, whereas TextDDI and DDI-GPT outperform other methods in scenarios involving distribution changes. To further analyse the reason for that result, we conduct a case study for the textual side information used and text processing outcomes of TextDDI (Zhu *et al.* 2023) in Table 3. TextDDI’s raw data include crucial drug information, such as names and interactions, adding realistic semantics to DDI prediction. The textual descriptions of two query drugs contain transferable pharmacological knowledge, like Aclidinium’s bronchodilatory activities and Butylscopolamine’s treatment and related receptors. This knowledge is not provided in other side information types, like drug fingerprints and biomedical networks. However, the DDI classifier struggles with irrelevant and distracting information in the raw data. The LLM that TextDDI uses effectively addresses this issue with its strong text comprehension and processing abilities. The experimental results show that LLM model in TextDDI obtains key pharmacological knowledge through textual side information, filter out irrelevant information, and provide useful data for DDI prediction. With TextDDI’s processed prompt, the DDI classifier can make accurate predictions.

**Table 3.** A case study on textual side information used and text process outcomes of TextDDI.

Raw textual data	**Aclidinium**: Aclidinium does not prolong the QTc interval or have significant effects on cardiac rhythm. Aclidinium bromide inhalation powder is *indicated for the long-term, maintenance treatment of bronchospasm associated with chronic obstructive pulmonary disease (COPD)*, including chronic bronchitis and emphysema. It *has a much higher propensity to bind to muscarinic receptors than nicotinic receptors*. FDA approved on 24 July 2012. Prevention of acetylcholine-induced bronchoconstriction effects was dose-dependent and lasted longer than 24 h. **Butylscopolamine**: Used to treat abdominal cramping and pain. Scopolamine butylbromide *binds to muscarinic M3 receptors* in the gastrointestinal tract. The inhibition of contraction reduces spasms and their related pain during abdominal cramping. Prediction: We can predict that the drug–drug interaction between Aclidinium and Butylscopolamine is that: Aclidinium may **decrease the bronchodilatory activities** of Butylscopolamine
Prompt processed by TextDDI	**Aclidinium**: Prevention of acetylcholine-induced bronchoconstriction effects was dose-dependent and lasted longer than 24 h. Aclidinium is a long-acting, competitive, and reversible anticholinergic drug, i.e. specific for the acetylcholine muscarinic receptors. It binds to all five muscarinic receptor subtypes to a similar affinity. Aclidinium’s effects on the airways are mediated through the M3 receptor at the smooth muscle to cause bronchodilation. **Butylscopolamine**: This prevents acetylcholine from binding to and activating the receptors which would result in contraction of the smooth muscle. Scopolamine butylbromide binds to *muscarinic M3 receptors* in the gastrointestinal tract. The inhibition of contraction reduces spasms and their related pain during abdominal cramping. Prediction: We can predict that the drug–drug interaction between Aclidinium and Butylscopolamine is that: Aclidinium may **increase the anticholinergic activities** of Butylscopolamine

The descriptions in “Raw textual data” part with italic script contain pharmacological knowledge. The words marked with underlines are important information relevant to DDI prediction of the two query drugs.

### 5.3 Performance analysis for different DDI types

Different DDI types can vary widely in their mechanism and they may be affected differently by realistic distribution changes in DDI prediction. Here, we conduct experiments to analyse the performance of DDI prediction methods for different DDI types. We select the best-performed methods from five DDI prediction categories as representatives, including MLP, MSTE, EmerGNN, TIGER, and DDI-GPT. Part of results on S1 task on Drugbank dataset are shown in Table 4, where we sample three major DDI types, three DDI types with medium frequency, and three long-tail DDI types.

**Table 4.** DDI prediction performance for different DDI types on Drugbank (S1 task).

Method	Distribution change	Major			Medium			Long-tail		
		#48	#46	#72	#29	#71	#57	#24	#1	#18
MLP	w/o	84.9	56.9	64.2	25.6	64.1	32.6	27.1	0.0	0.0
	w	73.9	52.3	**44.5**	0	17.5	13.2	15.1	0.0	0.0
MSTE	w/o	80.0	65.5	52.3	16.4	24.0	30.4	0.0	22.2	0.0
	w	74.9	**63.2**	37.9	2.9	10.9	0.3	9.9	17.1	0.0
EmerGNN	w/o	82.4	73.0	59.3	77.2	96.7	86.0	59.1	83.3	75.0
	w	65.3	59.1	40.8	61.8	24.1	56.7	35.1	55.7	35.0
TIGER	w/o	75.8	60.4	55.4	46.7	93.8	95.9	27.6	53.7	62.5
	w	73.5	42.5	37.9	32.3	56.1	**87.4**	5.6	48.5	13.3
DDI-GPT	w/o	82.5	69.5	50.7	80.7	92.4	89.2	54.4	66.7	65.0
	w	**76.1**	62.3	42.3	**67.7**	**72.4**	84.3	**58.3**	**61.4**	**53.3**

Here, “Major”, “Medium”, “Long-tail” denote DDI types with high, medium, low occurrence frequency, respectively. “w/o” and “w” denote the setting without and with distribution change introduced. For each DDI type, the best results for “w/o” and “w” setting are marked by underline and **bold**, respectively.

Generally, MLP and MSTE can achieve good performance on major DDI types, but they perform poorly on medium and long-tail DDI types. This suggests that these methods largely rely on sufficient labeled training data for prediction. Compared with two methods above, EmerGNN, TIGER, and DDI-GPT can achieve better performance on medium and long-tail DDI types. When distribution changes are introduced, EmerGNN and TIGER have a significant performance drop on all of DDI types. DDI-GPT significantly outperforms other methods on medium and long-tail DDI types with distribution changes introduced, indicating that LLMs can effectively handle the problem of distribution changes and lack of labeled training data for emerging DDI prediction.

### 5.4 Controlling distribution change

Here, we tune the parameter γ (introduced in Section 3.3) to control distribution changes between drug sets and analyse the performance of existing DDI methods with different normalized γ values. Similar as Section 5.3, we evaluate representative methods from each of the five categories.

**Figure 7.**
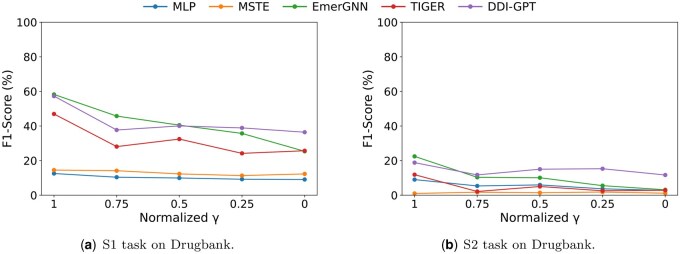
Tuning γ in the setting with distribution change for DDI prediction on Drugbank dataset.

The experiment results on Drugbank dataset are demonstrated in Fig. 7. With the decrease of the parameter γ, the distribution changes between training and test DDIs also become more significant. The performances of MLP and MSTE are unsatisfactory compared with other methods. For the other three methods, their performance drops significantly with distribution change becoming more significant, suggesting their sensitivity to distribution changes. Also, among the existing DDI methods, DDI-GPT performs relatively better than other methods when distribution changes are more significant. This suggests that the prior knowledge in LLMs and the textual side information used in DDI-GPT can help alleviate the negative impact of distribution changes on DDI prediction performance.

## 6 Related works

### 6.1 DDI prediction

The field of DDI prediction has attracted increasing attention in recent years and different types of techniques have been proposed. Feature-based methods (Rogers and Hahn 2010, Ryu *et al.* 2018, Liu *et al.* 2022) mainly rely on drug features and design a classifier to map the features of drug pairs to DDI types. Since data of drug interactions can be naturally represented as a graph, graph learning methods have been widely used to predict DDIs. Embedding-based methods (Karim *et al.* 2019, Yao *et al.* 2022) follow the idea of knowledge graph embedding learning, which design a model to learn embeddings of drugs and interactions and uses the embeddings to measure the interactions. GNN-based methods (Zitnik *et al.* 2018, Lin *et al.* 2021, Yu *et al.* 2021, Zhang *et al.* 2023, Wang *et al.* 2024) utilize GNNs to encode structure information from DDI or molecular graphs for DDI prediction. Graph transformer-based methods (Chen *et al.* 2024, Su *et al.* 2024) introduce graph transformer to better capture global structural information from drug-related structural knowledge. LLM-based methods (Zhu *et al.* 2023, Xu *et al.* 2024) utilize a large language model to predict DDI based on descriptions of drugs and relations.

### 6.2 Simulation of distribution changes in machine learning

Distribution changes commonly exist when deep learning methods are used in real-world scenarios, where the training and test data follow different distributions. They typically stem from factors such as covariate shift in input features, sampling bias in data collection, or concept drift in dynamic systems. To systematically study this phenomenon, existing works often simulate distribution changes. For example, in computer vision, it is common to use images captured under varied conditions as source and target datasets, thereby simulating distribution shifts encountered in real-world scenarios (Glorot *et al.* 2011). There are also works that sample long-tail data, so as to simulate the distribution changes (Jamal *et al.* 2020). Recently, strategies that simulate distribution changes have been introduced into bioinformatics to better capture realistic scenarios. Lo-Hi (Steshin 2023) proposes drug split strategies to simulate lead optimization and hit identification tasks in real drug discovery process. SPECTRA (Ektefaie *et al.* 2024) evaluates model generalizability by plotting performance against decreasing cross-split overlap and reporting the area under this curve. DataSAIL (Joeres *et al.* 2025) models data split as a combinatorial optimization problem and find leakage-reduced data splits based on optimization strategies.

## 7 Conclusion and future works

In this work, we propose DDI-Ben, a benchmark for DDI prediction in a perspective from drug distribution changes. We propose a distribution change simulation framework, i.e. compatible with different drug split strategies to reflect distribution changes in real-world emerging DDI prediction problems. Through extensive benchmarking evaluation, we find that the distribution changes can cause significant performance drops for existing DDI methods and LLM-based methods exhibiting superior robustness. To facilitate future research, we release benchmark datasets that include simulated distribution changes. For future work, designing LLM-based methods can potentially alleviate the negative impact of distribution changes in emerging DDI prediction. Domain adaptation methods can also be a potential research direction for practical solutions to deal with distribution changes in real-world emerging DDI prediction.

## Supplementary Material

btaf569_Supplementary_Data

## Data Availability

Our study used open-access datasets, and these DDI datasets and the source code of DDI-Ben are available in the GitHub repository as follows: https://github.com/LARS-research/DDI-Bench.
