# Supplementary material for: Benchmarking drug–drug interaction prediction methods: a perspective of distribution changes
Source: Bioinformatics. 2025 Oct 14;41(11):btaf569. doi: 10.1093/bioinformatics/btaf569 (PMC12579548; doi:10.1093/bioinformatics/btaf569)
Supplement: btaf569_Supplementary_Data [file btaf569_supplementary_data.pdf]

A. Supplementary Information of DDI-Ben

A.1. Detailed Information of Drug Clusters in Real-world Data

In Table 5, we provide the detailed information of drug clusters in Figure 4, including the drug names and explanations for the clusters.

Table 5. Detailed information of drug clusters in Figure 4.

Time period	1980-1989	1990-1999	2010-2019
Clustering reasons	new drug safety issues	technological breakthrough	new epidemics
Drugs in the cluster	Ibuprofen, Flurbiprofen, Fenoprofen	Ramipril, Benazepril, Moexipril, Fosinopril, Captopril, Perindopril, Quinapril, Trandolapril	Daclatasvir, Ledipasvir, Elbasvir, Pibrentasvir, Velpatasvir
Explanation	Severe gastrointestinal side effects of early NSAIDs (e.g., aspirin-induced ulcers) prompted the 1980s development of safer alternatives like Ibuprofen, Fenoprofen and Flurbiprofen, which inhibit COX enzymes to alleviate inflammation with reduced toxicity.	Breakthroughs in RAAS pathophysiology led to 1990s ACE inhibitors (Ramipril, Benazepril, Moexipril, Fosinopril, Captopril, Perindopril, Quinapril and Trandolapril) that suppress angiotensin II, clinically proven to protect hearts and kidneys through targeted vascular regulation.	Hepatitis C virus (HCV)-induced liver cirrhosis and cancer drove the development of NS5A inhibitors, including Daclatasvir, Pibrentasvir, Ledipasvir, Velpatasvir, and Elbasvir to directly block viral replication.

A.2. Similarity Measurement for Cluster-based Drug Split

In this work, we utilize Tanimoto Coefficient between the fingerprints of two drugs as the similarity measurement $S(\cdot,\cdot)$ of drug pairs:

$$S(u,v) = \frac{f(u)^\mathsf{T} f(v)}{||f(u)||^2 + ||f(v)||^2 - f(u)^\mathsf{T} f(v)} \tag{1}$$

where u, v are drugs and $f(\cdot)$ is the fingerprint of a drug. Actually, the Tanimoto Coefficient is a widely used similarity measurement between drugs in pharmacy.

A.3. Calculation of Consistency Index in Section 3.4

Here we present the calculation strategy of consistency index mentioned in Section 3.4 in Algorithm 2. Note that we denote the approval time of a certain drug u as y_u. We use a penalty value P_i to measure each error in drug split, and we assume that the larger the distance, the more severer the split error is.

Algorithm 2 Calculation of consistency index.

Require: The drug split result of 8 drug split scheme as known drug sets $D_k^i (i = 1, \ldots, 8)$ and new drug sets $D_n^i (i = 1, \ldots, 8)$. Threshold year for realistic drug split scheme as y_t.

Based on the threshold year y_t, split drugs with earlier approval time than y_t into known drug set D_k and drugs with later approval time than y_t into new drug set D_n, which is the realistic drug split scheme.
for $i = 1, \ldots, 8$ **do**
  $P_i = 0$. // Initialize the penalty value
  for each drug $u \in D_k^i \cup D_n^i$ **do**
    if $(u \in D_k^i \wedge u \in D_n) \vee (u \in D_n^i \wedge u \in D_k)$ **then**
      $P_i = P_i + |y_u - y_t|$.
    end if
  end for
end for
for $i = 1, \ldots, 8$ **do**
  $C_i = \frac{\max_{i=1}^{8}\{P_i\}}{P_i}$.
end for
return Consistency index $C_i (i = 1, \ldots, 8)$.

A.4. Significance Test for Consistency Index Comparison among Different Split Strategies

Figure 8 demonstrates the significance test for consistency index comparison among different data split strategies, which verifies that the proposed cluster-based split is more consistent with real-world data split.

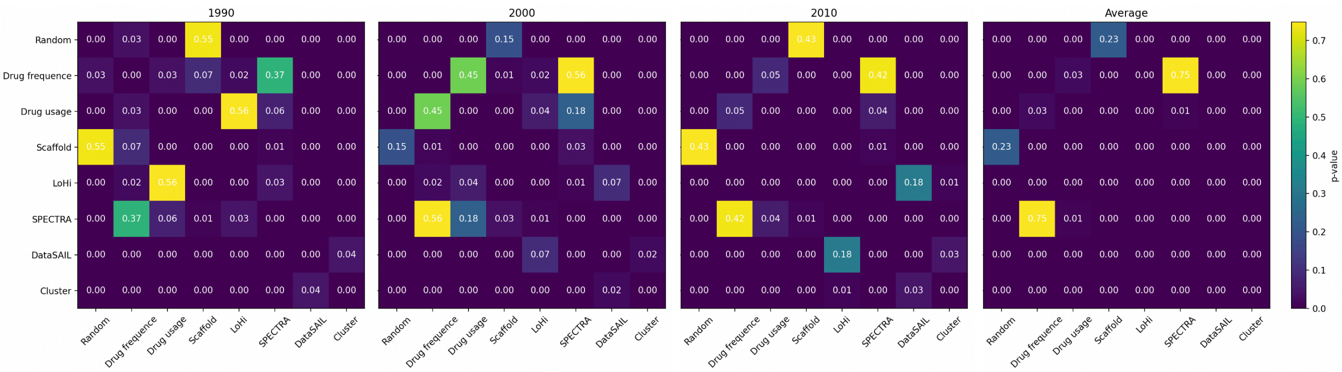

Fig. 8: Significance test for consistency index comparison.

A.5. Consistency Index Comparison among different similarity threshold γ for cluster-based split

Here we provide the consistency index with real-world drug split among different different similarity threshold γ for cluster-based split. We can see that setting the normalized similarity threshold γ as 1 could achieve highest consistency with realistic drug split scheme.

A.6. Statistics of Datasets.

Here we provide the general statistics of the two datasets used in experiments in Table 6. Here $\mathcal{V}_{\text{DDI}}$ denotes the set of drugs, $\mathcal{R}_{\text{DDI}}$ denotes the set of drug-drug interaction types, and $\mathcal{N}_{\text{DDI}}$ denotes the set of DDI triplets.

Table 6. General statistics of two datasets.

Dataset	$\lvert\mathcal{V}_{\text{DDI}}\rvert$	$\lvert\mathcal{R}_{\text{DDI}}\rvert$	$\lvert\mathcal{N}_{\text{DDI}}\rvert$
Drugbank	1,710	86	188,509
TWOSIDES	645	209	116,650

A.7. Summary of Existing DDI methods.

Our summary of existing DDI prediction methods is shown in Table 7.

A.8. Supplementary Information of Evaluation Metrics.

According to evaluation metric mentioned in Section 4.3, the evaluation metrics include F1-Score, accuracy and Cohen's Kappa for Drugbank:

- F1-Score (Macro) $= \frac{1}{||\mathcal{P}_D||}\sum_{p\in\mathcal{P}_D}\frac{2P_p\cdot R_p}{P_p+R_p}$, where P_p and R_p are the precision and recall for the interaction type p, respectively.
- Accuracy: the proportion of correctly predicted interaction types relative to the ground-truth interaction types.
- Cohen's Kappa: $\kappa = \frac{A_p - A_e}{1 - A_e}$, where A_p is the observed accuracy and A_e is the probability of randomly seeing each class.

And ROC-AUC, PR-AUC and accuracy for TWOSIDES:

- ROC-AUC $= \sum_{k=1}^{n} TP_k \Delta FP_k$ measures the area curve of receiver operating characteristics. TP_k and FP_k are the true positive rate and false positive rate at the k-th operating point.
- PR-AUC $= \sum_{k=1}^{n} P_k \Delta R_k$ measures the area under curve of precision-recall. Here P_k and R_k are the precision and recall at the k-th operating point.
- Accuracy: the proportion of correctly predicted DDIs for each DDI type.

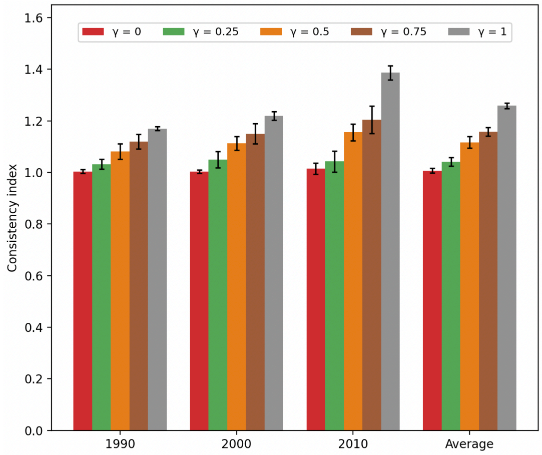

Fig. 9: Figure for time threshold of the realistic drug split scheme w.r.t the consistency index of different similarity threshold γ for cluster-based split.

A.9. Model Training for Methods in DDI-Ben

All the experiments in this work are conducted on a 24GB NVIDIA GeForce RTX 4090 GPU for 200 epochs. To obtain the best performance of existing computational DDI methods, we conduct comprehensive hyper-parameter tuning for each method. Table 8 shows a comprehensive list of hyper-parameters for each method, where we reference the tunning space of the original works. Hyperparameter tunning is conducted via Bayesian optimization method.

A.10. The Meaning of Selected DDI Types in Section 5.3 in Drugbank Dataset

- #48: The risk or severity of adverse effects can be increased when #Drug1 is combined with #Drug2.
- #46: The metabolism of #Drug2 can be decreased when combined with #Drug1.
- #72: The serum concentration of #Drug2 can be increased when it is combined with #Drug1.
- #29: #Drug1 may increase the orthostatic hypotensive activities of #Drug2.
- #71: #Drug1 may decrease the excretion rate of #Drug2 which could result in a higher serum level.
- #57: #Drug1 may decrease the cardiotoxic activities of #Drug2.
- #24: #Drug1 may increase the atrioventricular blocking (AV block) activities of #Drug2.
- #1: #Drug1 may increase the photosensitizing activities of #Drug2.
- #18: #Drug1 may increase the vasoconstricting activities of #Drug2.

A.11. Construction of Real-world Emerging DDI Prediction Dataset

In this work, we use the Drugbank dataset to construct the real-world emerging DDI prediction dataset. We first extract 1710 drugs and their names from the DDI dataset. Then we collect the approval times of these drugs based on their names from FDA Drugs Database `https://www.drugfuture.com/fda/`. Among these drugs, totally 886 drugs have available approval time information, with their distribution shown in Figure 10.

A.12. Limitations of DDI-Ben

In DDI-Ben, we emphasize the importance of distribution changes that could greatly affect the performance of emerging DDI prediction performance in realistic drug development scenarios. Although we provide an ensembling method of best-performing existing DDI prediction techniques to handle the negative impact of distribution changes, its incremental gains remain notably limited. New methods that can effectively improve the performance of DDI prediction under distribution changes are still needed, which is also the future work of this paper.

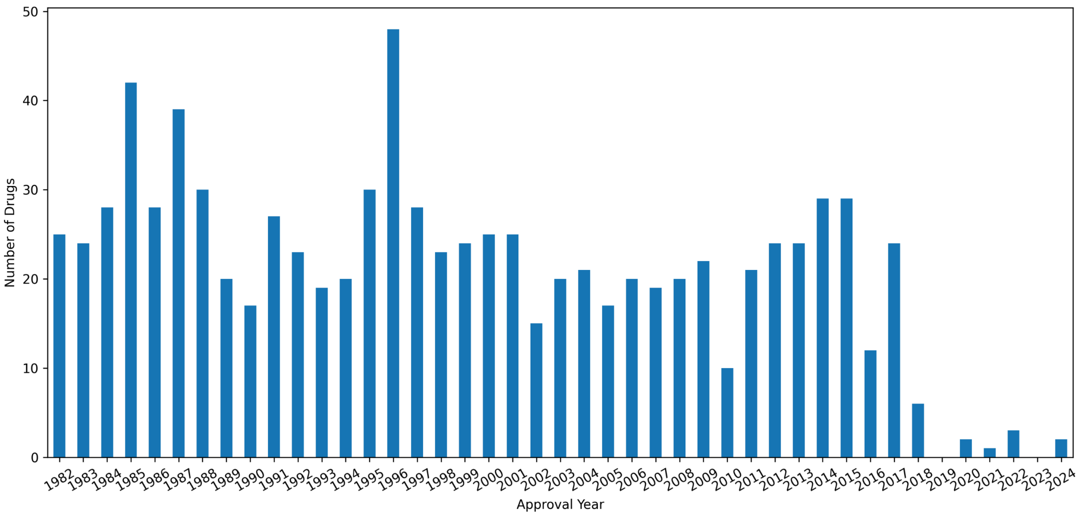

Fig. 10: The distribution of available approval time of drugs in Drugbank dataset.

Table 7. Summary of Drug-Drug Interaction Prediction Methods.

Method	Categorization	Description	Availability
MLP	Feature based	Uses drug fingerprints to compute predicted logits.	×
DeepDDI	Feature based	Takes drug chemical structures and names as inputs, predicting DDI types via DNN.	https://bitbucket.org/kaistsystemsbiology/deepddi/src/master/
SFLLN	Feature based	Integrates four drug features and uses linear neighborhood regularization for DDIs.	https://bitbucket.org/kaistsystemsbiology/deepddi/src/master/
DDIMDL	Feature based	Constructs sub-models from drug features and combines them for DDI prediction.	https://github.com/BioMedicalBigDataMiningLabWhu/SFLLN.SFLLN
CSMDDI	Feature based	Uses RESCAL-based method to obtain drug and DDI type representations, learning a mapping function.	https://github.com/itsosy/csmddi
AMDE	Feature based	Uses graph and sequential encoders for drug features, with a multi-dimensional decoder for DDIs.	https://github.com/wan-Ying-Z/AMDE-master
ComplEx	Embedding based	Maps entities and relations into a complex vector space to calculate DDI probabilities.	×
Graph-Embedding-4DDI	Embedding based	Applies RDF2Vec, TranE, and TransD to extract drug feature vectors.	https://github.com/rcelebi/GraphEmbedding4DDI/
KG-DDI	Embedding based	Uses KG embedding methods and a Conv-LSTM network to predict DDI relations.	https://github.com/rezacsedu/Drug-Drug-Interaction-Prediction
MSTE	Embedding based	Learns drug and relation embeddings, designing a score function for prediction.	https://github.com/galaxysunwen/MSTE-master
DDKG	Embedding based	Learns global drug representations using neighboring embeddings and triple facts.	https://github.com/Blair1213/DDKG
Decagon	GNN based	Uses drugs, genes, and diseases with GCN to update drug representations and predict DDIs.	https://github.com/mims-harvard/decagon
SkipGNN	GNN based	Constructs a skip graph to obtain node embeddings, predicting DDIs via a decoder.	https://github.com/kexinhuang12345/SkipGNN
KGNN	GNN based	Encodes drug and neighborhood info using GNN to predict DDIs.	https://github.com/xzenglab/KGNN
SSI-DDI	GNN based	Models drug molecular graphs and predict DDI based on interaction between substructure of query drug pairs.	https://github.com/kanz76/SSI-DDI
SumGNN	GNN based	Uses GNN to compute subgraph representations from an augmented network for DDI prediction.	https://github.com/yueyu1030/SumGNN
DeepLGF	GNN based	Fuses local chemical structure, global, and biological function info for DDI prediction.	https://github.com/MrPhil/DeepLGF
MRCGNN	GNN based	Multi-relation graph contrastive learning strategy to better characteristics of rare DDI types.	https://github.com/Zhankun-Xiong/MRCGNN
EmerGNN	GNN based	Uses flow based GNN with attention to update drug representations for DDIs.	https://github.com/yzhangee/EmerGNN
KnowDDI	GNN based	Optimizes drug embeddings from augmented subgraphs to predict DDIs.	https://github.com/LARS-research/KnowDDI
SAGAN	GNN based	Utilizes a transfer learning strategy to enhance the cross-domain generalization ability of GNNs.	https://github.com/wyx2012/SAGAN
KSGTN-DDI	Graph-transformer based	Uses a Key Substructure-aware Graph Transformer for DDI prediction.	×
TIGER	Graph-transformer based	Uses a Transformer based framework with self-attention and dual-channel network for DDI prediction.	https://github.com/Blair1213/TIGER
DrugDAGT	Graph-transformer based	Uses dual-attention graph transformer with contrastive learning for DDI prediction.	https://github.com/codejiajia/DrugDAGT
TextDDI	LLM based	Designs an LM-based predictor with RL-based selector for short DDI descriptions.	https://github.com/zhufq00/DDIs-Prediction
DDI-GPT	LLM based	Uses knowledge graphs and pre-trained models to capture contextual dependencies for DDI prediction.	https://github.com/Mew233/ddigpt
K-Paths	LLM based	Design a retrieval framework that extracts meaningful paths from KGs, enabling LLMs to predict unobserved drug-drug interactions.	https://github.com/rsinghlab/K-Paths

Table 8. Hyperparameter search space for all compared methods.

Method	Hyperparameter	Search Space
General Settings	Learning rate	[0.0001, 0.0003, 0.001, 0.003]
	Weight decay	[1e-6, 1e-5, 1e-4, 0]
	Dropout rate	[0, 0.1, 0.2, 0.3, 0.4, 0.5]
	Batch size	[64, 128, 256]
	Training epoch	[100]
MLP	Layer number	[1, 2, 3]
	Hidden dimension	[50, 100, 200]
MSTE	Embedding dimension	[50, 100, 200]
Decagon	Layer number	[1, 2, 3]
	Hidden dimension	[50, 100, 200]
SSI-DDI	Hidden dimension	[32, 64]
MRCGNN	Hidden dimension	[32, 64]
EmerGNN	Subgraph sampling hop	[1, 2, 3, 4]
	Hidden dimension	[32, 64]
SAGAN	Hidden dimension	[32, 64]
TIGER	Layer number	[1, 2, 3]
	Hidden dimension	[32, 64]

B. Additional Experimental Results

B.1. Additional Experimental Results for General Method Performance

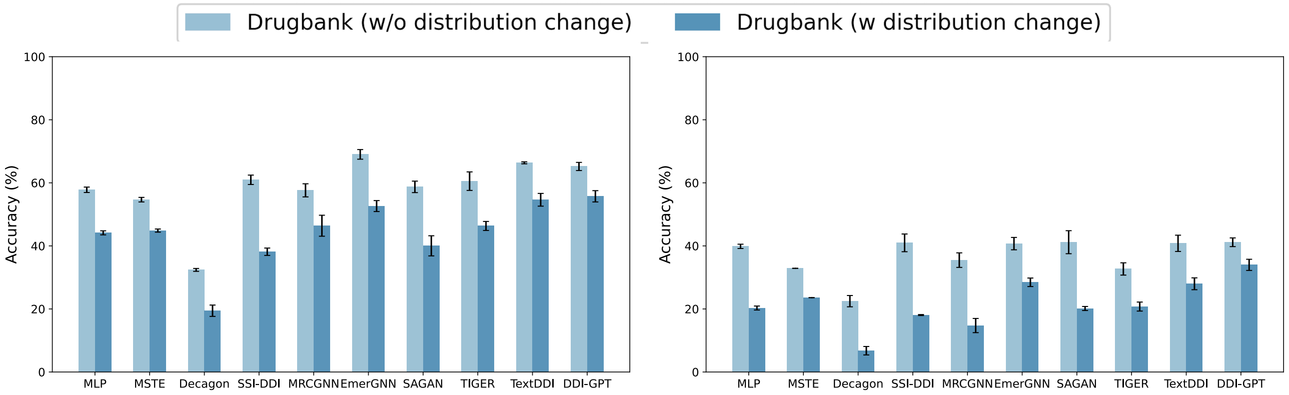

(a) Accuracy in S1 task on Drugbank. (b) Accuracy in S2 task on Drugbank.

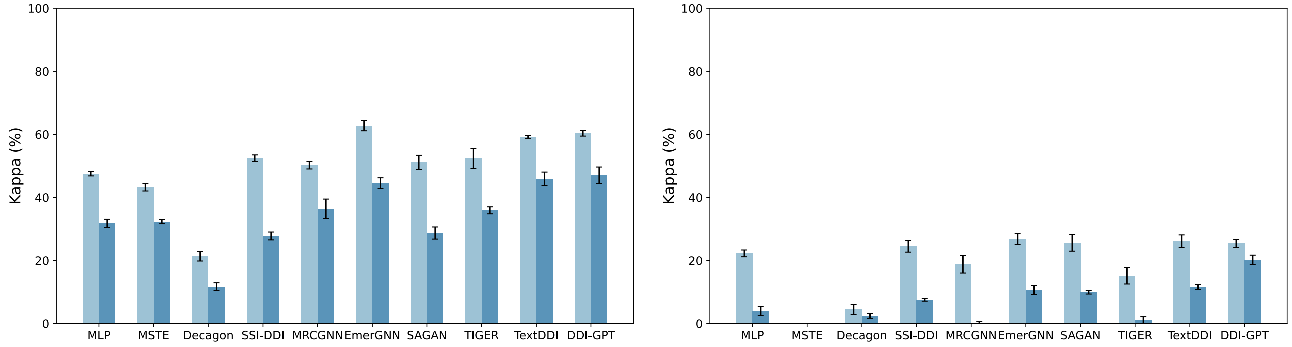

(c) Cohen's Kappa in S1 task on Drugbank. (d) Cohen's Kappa in S2 task on Drugbank.

Fig. 11: Performance comparison in the setting with and without distribution change in S1-S2 tasks. Here is the results that use accuracy and Cohen's Kappa as evaluation metrics for Drugbank.

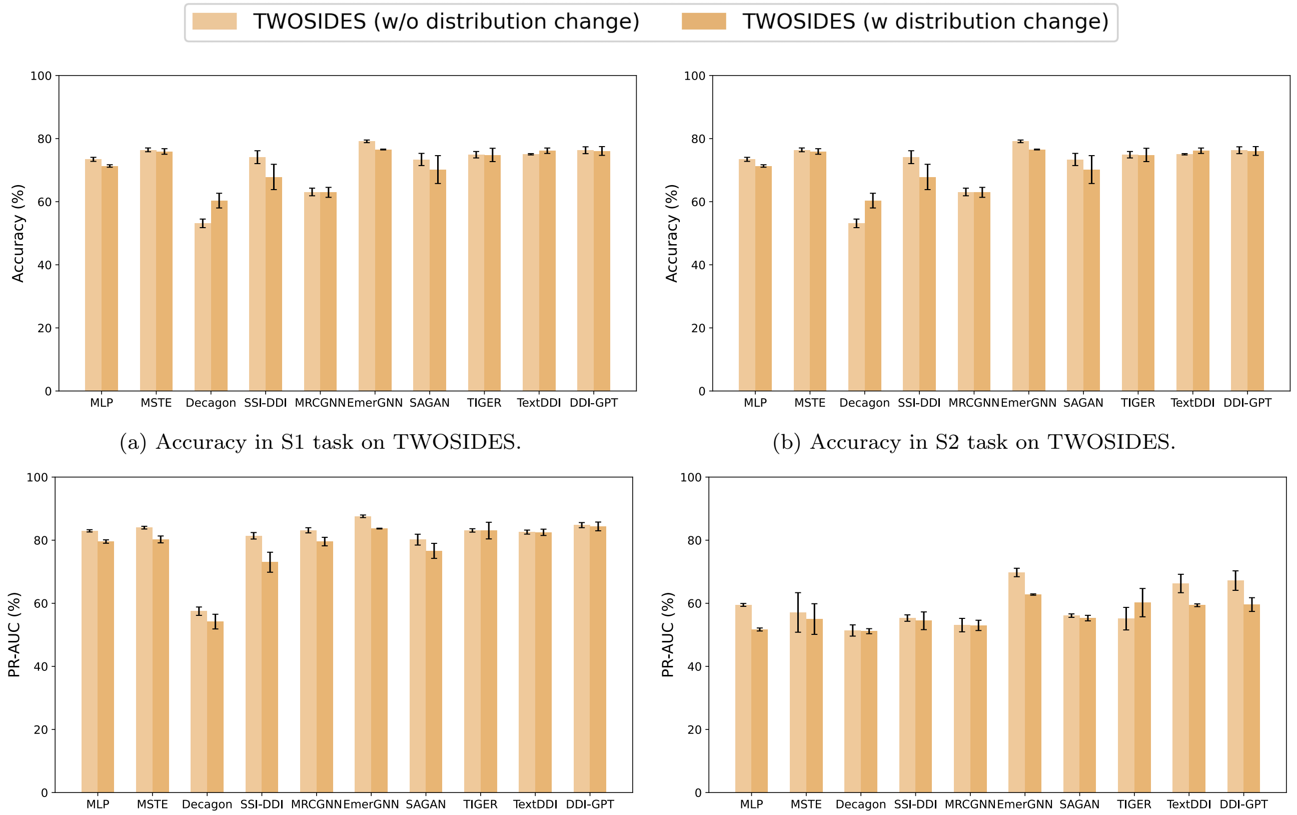

(a) Accuracy in S1 task on TWOSIDES.

(b) Accuracy in S2 task on TWOSIDES.

(c) PR-AUC in S1 task on TWOSIDES.

(d) PR-AUC in S2 task on TWOSIDES.

Fig. 12: Performance comparison in the setting with and without distribution change in S1-S2 tasks. Here is the results that use PR-AUC and accuracy as evaluation metrics for TWOSIDES.

B.2. Relative Performance Comparison of Different Methods on Drugbank and TWOSIDES Datasets

Table 9. Comparison of different methods on Drugbank dataset **without** and **with** distribution change. "Emb" is short for "Embedding"; "GT" is short for "Graph-transformer". The evaluation metrics are presented in percentage (%) with best values in boldface. Avg. Score is the mean of the scores across two settings (S1 and S2) for each method, representing overall performance. Rank is determined based on the Avg. Score, with the highest Avg. Score receiving rank 1.

Datasets		**Drugbank (F1-Score, w/o distribution change)**				**Drugbank (F1-Score, w distribution change)**			
Category	Methods	S1	S2	Avg. Score	Rank	S1	S2	Avg. Score	Rank
Feature	MLP	12.5±2.3	9.0±1.9	10.8	8	9.0±2.1	2.8±0.6	5.9	9
Emb	MSTE	14.5±0.7	1.0±0.0	7.8	9	12.3±0.3	1.2±0.0	6.8	8
GNN	Decagon	11.6±0.5	2.9±0.0	7.3	10	4.1±0.0	1.3±0.1	2.7	10
	SSI-DDI	50.5±1.8	19.1±1.3	34.8	5	19.1±0.1	6.5±0.5	12.8	6
	MRCGNN	41.7±1.3	8.6±1.6	25.2	7	12.0±2.8	2.3±0.9	7.1	7
	EmerGNN	56.9±1.7	**22.5±1.2**	**39.7**	1	34.0±2.1	3.1±0.3	18.6	3
	SAGAN	51.3±0.6	18.8±2.2	35.0	4	22.3±2.6	8.1±1.3	15.2	4
GT	TIGER	47.0±2.5	11.9±2.0	29.5	6	25.7±1.4	2.9±1.2	14.3	5
LLM	TextDDI	56.8±0.8	18.2±0.2	37.5	3	**36.7±1.0**	10.9±1.0	23.8	2
	DDI-GPT	**57.3±1.4**	18.8±2.2	38.0	2	36.4±1.9	**11.6±1.8**	**24.0**	1

Table 10. Comparison of different methods on TWOSIDES dataset **without** and **with** distribution change.

Datasets		**TWOSIDES (ROC-AUC, w/o distribution change)**				**TWOSIDES (ROC-AUC, w distribution change)**			
Category	Methods	S1	S2	Avg. Score	Rank	S1	S2	Avg. Score	Rank
Feature	MLP	84.7±0.2	60.0±0.6	72.4	4	78.8±0.5	47.1±0.6	63.0	9
Emb	MSTE	86.1±0.3	57.0±6.5	71.4	5	82.4±1.0	52.7±7.0	67.6	5
GNN	Decagon	59.3±1.7	49.5±2.8	54.4	10	57.5±2.1	50.8±0.8	54.2	10
	SSI-DDI	81.5±1.3	55.6±0.9	68.5	7	74.4±3.8	54.7±2.4	64.5	8
	MRCGNN	83.4±0.4	53.2±1.9	68.3	8	79.8±1.1	53.4±1.4	66.6	6
	EmerGNN	**86.4±0.5**	**72.5±1.9**	**79.5**	1	84.8±0.2	**60.5±0.3**	72.7	2
	SAGAN	80.9±0.8	56.4±0.8	69.2	6	77.0±3.0	55.6±0.7	66.3	7
GT	TIGER	82.7±0.5	52.5±2.9	67.6	9	84.6±1.7	58.7±5.0	71.7	4
LLM	TextDDI	83.3±0.6	68.7±1.2	76.0	3	**85.5±0.7**	60.3±2.3	**72.9**	1
	DDI-GPT	85.2±0.7	68.5±2.8	76.8	2	85.0±1.6	60.4±1.9	72.7	2

B.3. Additional Results for Experiment on DDI Types

The results of DDI prediction performance for different DDI types on Drugbank in S2 task are shown in Table 11.

Table 11. DDI prediction performance for different DDI types on Drugbank (S2 task). Here "Major", "Medium", "Long-tail" denote DDI types with high, medium, low occurrence frequency, respectively. "w/o" and "w" denote the setting without and with distribution change introduced. For each DDI type, the best results for "w/o" and "w" setting are marked by underline and **bold**, respectively.

Method	Distribution change	Major			Medium			Long-tail		
		#48	#46	#72	#29	#71	#57	#24	#1	#18
MLP	w/o	74.5	22.9	<u>41.8</u>	16.7	59.3	22.2	11.1	28.6	16.7
	w	58.4	13.9	20.2	0.0	0.0	7.1	0.0	0.0	0.0
MSTE	w/o	<u>100.0</u>	0.0	0.0	0.0	0.0	0.0	0.0	0.0	0.0
	w	**100.0**	0.0	0.0	0.0	0.0	0.0	0.0	0.0	0.0
EmerGNN	w/o	67.0	<u>38.4</u>	34.1	<u>37.5</u>	85.2	<u>43.3</u>	<u>37.8</u>	<u>82.9</u>	0.0
	w	74.0	**41.2**	8.0	8.0	0.0	0.0	0.0	0.0	0.0
TIGER	w/o	65.5	16.7	22.4	16.7	<u>93.0</u>	22.2	7.4	28.6	50.0
	w	61.5	2.3	1.3	5.0	7.4	2.4	0.0	5.6	16.7
DDI-GPT	w/o	80.4	19.1	37.1	30.6	68.2	38.1	30.4	58.0	<u>66.7</u>
	w	66.2	13.7	**23.6**	**27.7**	**24.7**	**43.6**	**17.4**	**26.3**	**41.7**

The performance of representative DDI prediction methods on all DDI types in Drugbank dataset is shown in Figure 13. Here the frequency of different DDI types is shown on the right.

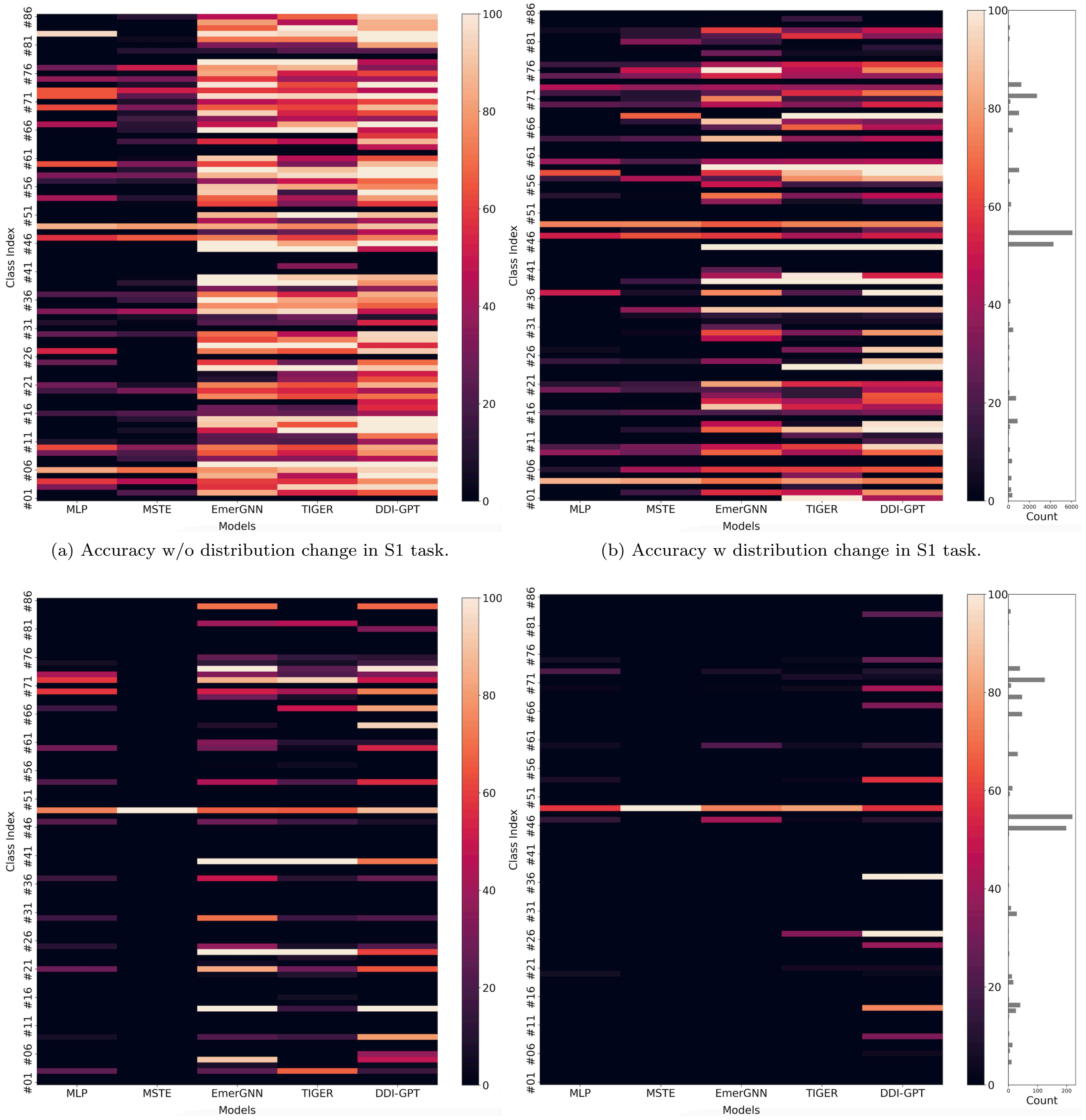

(a) Accuracy w/o distribution change in S1 task.

(b) Accuracy w distribution change in S1 task.

(c) Accuracy w/o distribution change in S2 task.

(d) Accuracy w distribution change in S2 task.

Fig. 13: The performance of representative DDI prediction methods for all DDI types on Drugbank dataset.

B.4. Additional Experimental Results for Controlling Distribution Change

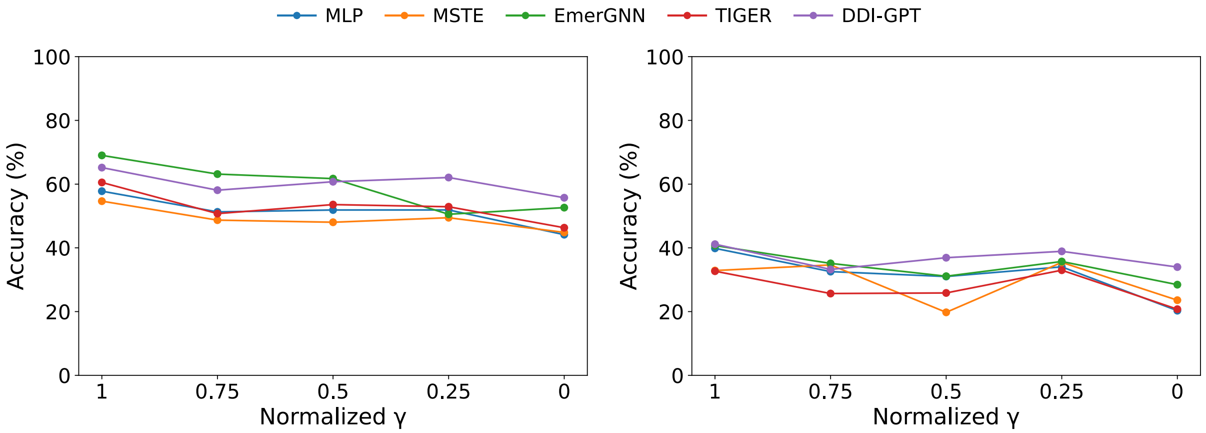

(a) Accuracy for S1 task on Drugbank.

(b) Accuracy for S2 task on Drugbank.

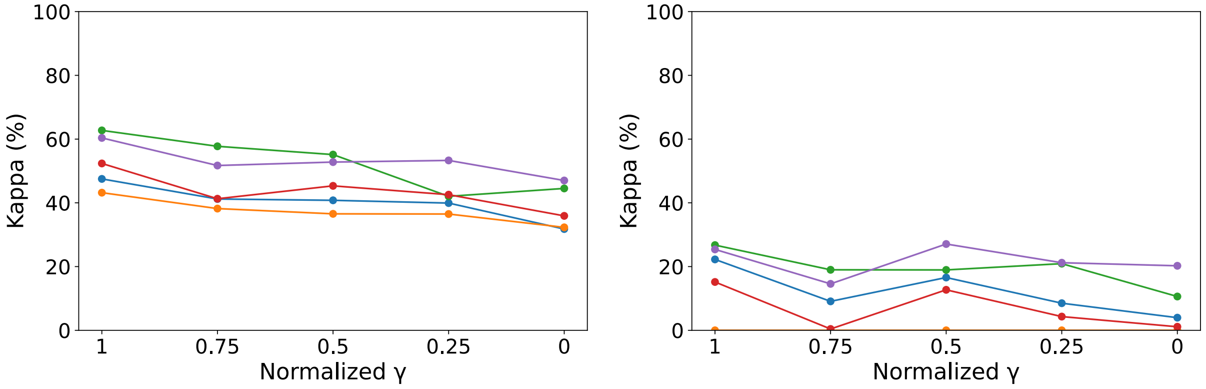

(c) Cohen's Kappa for S1 task on Drugbank.

(d) Cohen's Kappa for S2 task on Drugbank.

Fig. 14: Tunning γ in the setting with distribution change on Drugbank dataset (accuracy and Cohen's Kappa as evaluation metric).

B.5. Benchmarking Results Using DataSAIL to Conduct Drug Split

In consistency comparison among different drug split scheme in Section 3.4, we can see that DataSAIL achieves the second highest consistency index with realistic drug split scheme. Here we further conduct experiments using DataSAIL to conduct distribution change simulation and compare the results with the setting without distribution change. As shown in Figure 15, we can see that GNN based method (EmerGNN), graph transformer based method (TIGER) and LLM based methods (DDI-GPT) still achieve relatively better performance. The performance degradation of various methods under distribution changes remains substantial compared with the setting without distribution changes. LLM based method (DDI-GPT) still achieves the best performance when distribution changes are introduced. These findings highlight the importance of accounting for distribution changes in emerging DDI prediction and confirm that the proposed simulation framework is compatible with different drug split strategies.

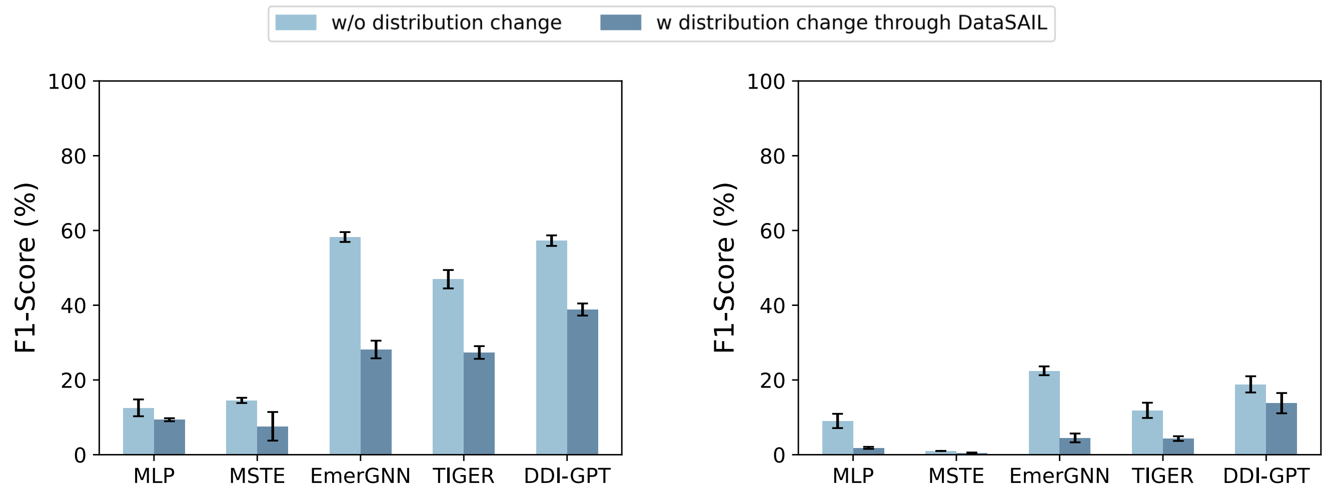

(a) S1 task on Drugbank.

(b) S2 task on Drugbank.

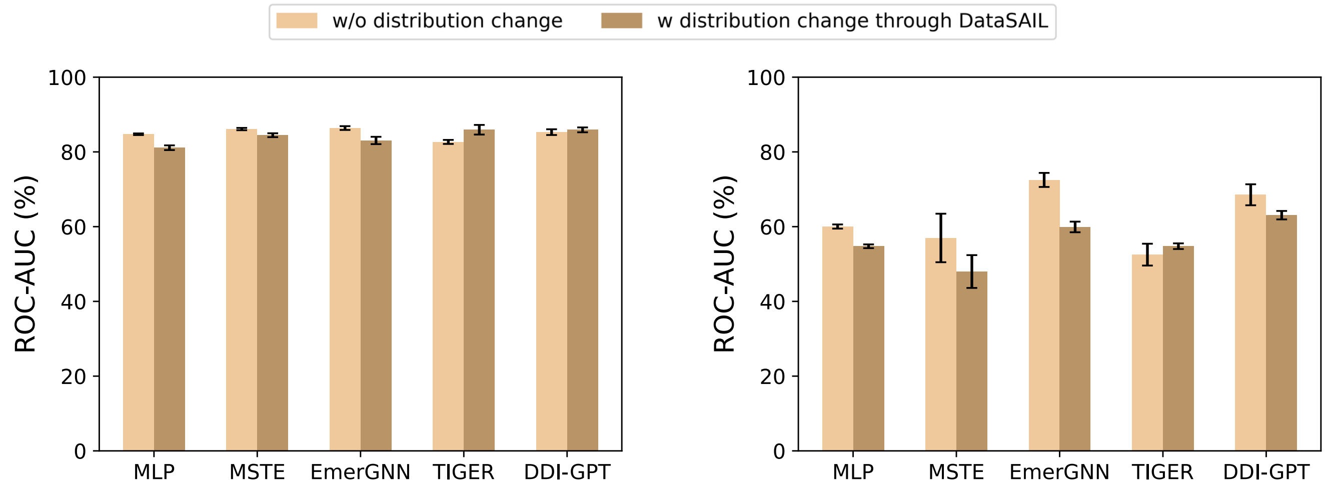

(c) S1 task on TWOSIDES.

(d) S2 task on TWOSIDES.

Fig. 15: Performance comparison for different types of DDI methods in the settings with and without distribution change in S1-S2 tasks. Here drug splitting for known and new drugs is conducted by DataSAIL and we utilize primary evaluation metric for each dataset (F1 for Drugbank and ROC-AUC for TWOSIDES).

B.6. Time and Memory Cost of Evaluated Methods

We provide the time and memory cost of all the evaluated methods in Table 12. These results are obtained by running the methods on the Drugbank dataset. We can see that generally GNN based methods, graph-transformer based method and LLM based method have more time and memory cost than feature based method and embedding based methods.

Table 12. Time and memory cost of the evaluated methods on Drugbank dataset.

Method	Time (min)	Memory cost (MB)
MLP	6	548
MSTE	16	640
Decagon	23	6736
SSI-DDI	49	3284
MRCGNN	836	3816
EmerGNN	725	7431
SAGAN	186	5819
TIGER	293	5734
TextDDI	1871	9404
DDI-GPT	1931	15356
